# Optimization of Neural Network Models of Computer Vision for Biometric Identification on Edge IoT Devices

**DOI:** 10.3390/jimaging11110419

**Published:** 2025-11-20

**Authors:** Bauyrzhan Belgibayev, Madina Mansurova, Ganibet Ablay, Talshyn Sarsembayeva, Zere Armankyzy

**Affiliations:** Department of Artificial Intelligence and Big Data, Faculty of Information Technology, Al-Farabi Kazakh National University, Almaty 050040, Kazakhstan; bauyrzhan.belgibaev@kaznu.edu.kz (B.B.); madina.mansurova@kaznu.edu.kz (M.M.); gans042004@gmail.com (G.A.); armankyzy_zere@kaznu.edu.kz (Z.A.)

**Keywords:** biometrics, identification, artificial intelligence, IoT, neural network, intelligent system, computer vision

## Abstract

This research is dedicated to the development of an intelligent biometric system based on the synergy of Internet of Things (IoT) technologies and Artificial Intelligence (AI). The primary goal of this research is to explore the possibilities of personal identification using two distinct biometric traits: facial images and the venous pattern of the palm. These methods are treated as independent approaches, each relying on unique anatomical features of the human body. This study analyzes state-of-the-art methods in computer vision and neural network architectures and presents experimental results related to the extraction and comparison of biometric features. For each biometric modality, specific approaches to data collection, preprocessing, and analysis are proposed. We frame optimization in practical terms: selecting an edge-suitable backbone (ResNet-50) and employing metric learning (Triplet Loss) to improve convergence and generalization while adapting the stack for edge IoT deployment (Dockerized FastAPI with JWT). This clarifies that “optimization” in our title refers to model selection, loss design, and deployment efficiency on constrained devices. Additionally, the system’s architectural principles are described, including the design of the web interface and server infrastructure. The proposed solution demonstrates the potential of intelligent biometric technologies in applications such as automated access control systems, educational institutions, smart buildings, and other areas where high reliability and resistance to spoofing are essential.

## 1. Introduction

The rapid development of digital technologies since the second decade of the 21st century has radically transformed traditional approaches to data collection, processing, and storage. As a result, intelligent systems based on artificial intelligence (AI) and Internet of Things (IoT) technologies have become integral to many domains. Biometric identification systems have gained particular relevance in the context of security and authentication, gradually replacing conventional methods that rely on passwords or physical access tokens.

Biometric technologies utilize inherent physiological and behavioral characteristics of individuals, providing higher accuracy, resistance to unauthorized use, and greater convenience for users. Among the various biometric modalities, face recognition and palm vein patterns have attracted significant attention due to their accuracy, robustness against spoofing attacks, and suitability for real-time applications [1,2]. Accordingly, this study focuses on the integration of facial feature analysis and palm vein recognition—while also acknowledging the broader potential of fingerprint and retinal structure analysis—as key components of reliable biometric authentication mechanisms [3]. However, the growing sophistication of digital forgeries, such as deepfakes and synthetic media, necessitates the adoption of advanced protection methods that go beyond surface-level analysis and leverage structural biometric information.

This work introduces the concept of an intelligent recognition system that combines two distinct biometric modalities with AI and IoT technologies to enhance security and spoof resistance in edge computing environments. The architecture integrates face recognition and palm vein analysis to create a hybrid biometric system.

The facial recognition module was implemented using the face_recognition library in Python (version 1.3.0) [4], augmented with stereo vision to generate depth maps. The resulting disparity information enables verification of physical presence, reducing the system’s vulnerability to photo- and video-based spoofing attacks. For face registration, a server-side prototype was developed with FastAPI and PostgreSQL, containerized using Docker, and deployed on Amazon Web Services (AWS). A lightweight administrative interface built with SQLAdmin provides efficient monitoring and system management. We do not consider fingerprints or retinal biometrics because they usually require contact-based sensors. Instead, we focus on contactless, hygienic modalities (face and palm-vein), which are better suited for IoT and edge deployments.

For recognition and identification, images from the open-source Contactless Knuckle, Palm Print and Vein Dataset [5] and VERA PalmVein dataset [6] were used. The preprocessing pipeline included median filtering, morphological normalization, binarization, and skeletonization to enhance the visibility of subcutaneous venous structures. As an initial attempt to address palm classification, convolutional neural network models based on VGG16 and ResNet50 were developed. Subsequently, a more advanced training strategy was employed: the ResNet50 model was fine-tuned using the Triplet Loss function. The objective was not merely to perform classification but to learn robust feature representations by maximizing inter-class separation in the embedding space.

The scientific contribution of this work lies in the development of a unified platform concept that integrates heterogeneous biometric signals—external (facial features) and internal (palm vein patterns)—into a single system. The incorporation of stereo vision further enhances system reliability by enabling the distinction between two-dimensional spoofing attacks with fake images and the genuine three-dimensional geometry of the human face.

The object of this research is intelligent biometric identification systems, while the subject encompasses AI- and IoT-based algorithms, deep neural network architectures, and the hardware–software components that enable their deployment on edge devices.

The expected outcome is a secure, scalable, and edge-compatible biometric authentication system that leverages AI and IoT to verify both physical presence and multimodal identity traits. This approach provides the foundation for the further advancement of multifactor authentication and intelligent access control systems. Edge computing plays a key role in modern biometric solutions by enabling decentralized, real-time decision-making directly at the data source [7,8]. Furthermore, the classification of artificial intelligence into narrow, general, and superintelligence has practical relevance for the implementation of embedded biometric applications [9].

The proposed solution has practical significance for domains such as secure access to banking services, turnstile-based campus entry control, employee attendance monitoring, patient identification in healthcare, and building access management. Its resilience against spoofing attempts using photos and videos constitutes a major advantage over traditional identification methods.

## 2. Related Works

Biometric recognition based on artificial intelligence and computer vision has been developing particularly actively in recent years, especially in the direction of enhancing system security and deploying models on devices with limited computational resources. Considerable attention has been devoted to neural network optimization, the integration of multimodal biometric features, and reducing vulnerability to spoofing attacks.

One of the earliest and most influential studies in the field of deep face recognition was conducted by Parkhi et al., who developed a convolutional neural network trained on large datasets that became the foundation for many modern face embedding models  [10]. Later, Zhang et al. introduced lightweight architectures such as MobileFaceNet, which achieve a balance between computational efficiency and recognition accuracy, making them suitable for mobile and embedded systems [11].

In the area of palm vein biometrics, Kumar and Zhou proposed a multi-line tracking method using near-infrared images, enabling the extraction of venous structures with high robustness against spoofing [12]. More recently, Hemis et al. explored the use of deep neural networks with triplet loss for palm vein image analysis, presenting a review of work that achieved higher verification accuracy than classical approaches [13].

Multimodal biometric systems are considered a promising way to improve reliability and robustness. For instance, Kartheek and Ramesh demonstrated that combining facial and fingerprint features using deep learning enhances recognition performance under varying conditions [14]. Their findings confirm the effectiveness of incorporating additional biometric modalities in applications that require a high degree of trustworthiness.

To counter presentation attacks, Määttä et al. proposed a liveness detection method based on texture analysis, which identifies fake faces by extracting micro-textural features from single images [15]. While this approach has proven effective against printed photo attacks, modern threats such as deepfakes demand more sophisticated solutions based on stereo vision and temporal modeling.

Regarding deployment on edge-class devices, Howard et al. presented the MobileNets architecture—an efficient convolutional model for mobile computer vision applications that has been widely adopted in edge-AI tasks [16]. Furthermore, Han et al. introduced a deep compression approach that combines pruning, quantization, and Huffman coding, significantly reducing neural network size and adapting models for devices with limited power consumption [17].

Taken together, these studies provide the foundation for designing secure, compact, and real-time biometric systems applicable to IoT environments with constrained computational capabilities.

## 3. Materials and Methods

### 3.1. System Architecture Overview

The proposed biometric system consists of two main subsystems: (1) a face recognition and liveness detection module based on stereo vision, and (2) a palm vein recognition module leveraging infrared imaging and convolutional neural networks. The entire platform is designed as a modular and scalable architecture. The server-side implementation is fully developed in Python, utilizing FastAPI for web services and SQLAlchemy for database interaction. All services are deployed in isolated Docker containers, ensuring portability and ease of maintenance. Our face recognition pipeline is based on the principles of deep metric learning described by King  [18], and is complemented by architectures such as ArcFace [19], DeepFace [20], and others implemented in open-source frameworks [21].

### 3.2. Face Recognition Performance

To counter photo and video replay attacks, the system includes a stereo camera module that captures depth information by computing disparity maps. This module consists of two UVC-compatible USB cameras (OV9732), mounted in parallel with a baseline of 7 cm, providing a resolution of 1280 × 720 at 30 fps, a lens base of 70 mm, and a field of view of 100 degrees. Furthermore, depth data streams can be combined with two-dimensional face embeddings, enabling adaptive decision thresholds. These thresholds are automatically tightened when depth noise increases, reducing the likelihood of false acceptances under challenging lighting conditions.

#### Stereo Camera Calibration, Depth Estimation, and Reprojection Error Analysis

The cameras were calibrated using the OpenCV stereo calibration module, based on 16 pairs of chessboard images with 10 × 7 inner corners and a square size of 25 mm. As a result of calibration, both intrinsic and extrinsic parameters were obtained and stored in .npz files. Disparity maps were computed using the StereoBM and StereoSGBM algorithms. Depth was estimated according to the following formula:

(1) $$Depth=\frac{f\times B}{d}$$

The overall reprojection error was measured at RMS = 0.464 px, which is considered a highly accurate result according to the criteria summarized in Table 1.

### 3.3. Liveness Metrics

The key indicators for liveness verification are the disparity variance (var ≈1432.4 for live faces vs. ≈26.5 for spoofed ones) and the depth difference between the nose and the face (≈855 pixels vs. ≈3.1 pixels). These metrics are computed in real time and compared against predefined thresholds. For visualization, grayscale, the Jet colormap, and  disparity map overlays are used.

### 3.4. Model Parameters

For the palm vein recognition model, we used the ResNet50 architecture, with an embedding dimension of 128. The Euclidean distance metric was used for comparing embeddings. The model was trained for 50 epochs with a learning rate of $1\times 10^{-4}$. Additionally, t-SNE visualization was applied to assess the quality of the learned embeddings, where the embeddings are represented in a 2D space, providing insight into class separability.

### 3.5. Algorithm for Palm Vein Recognition

To unify both biometric modalities, the complete training and evaluation process for face and palm vein recognition is summarized in Algorithm 1.
**Algorithm 1** Face and Palm Vein Recognition Procedure**Require:** Facial image dataset $D_{f}$, palm vein image dataset $D_{p}$ **Ensure:** Trained recognition models $M_{f}$, $M_{p}$, and stored embeddings
  1:Load datasets $D_{f}$ and $D_{p}$  2:Preprocess all samples: denoising, normalization, ROI extraction, and augmentation  3:**for** e=1 **to** *E* **do**  4:    **for all** mini-batch *B* in $\left\{D_{f},D_{p}\right\}$ **do**  5:        Forward propagate *B* through the corresponding network (face or palm)  6:        Compute loss *L* (Cross-Entropy or Triplet Loss)  7:        Backpropagate and update parameters using Adam  8:    **end for**  9:**end for**10:Evaluate on test sets: Accuracy, Precision, Recall, F1, FAR, FRR11:Store embeddings and metrics in the PostgreSQL database


### 3.6. Database Structure and Backend

Face embeddings (128-dimensional vectors) are computed using the face_recognition Python library, which leverages a pre-trained ResNet convolutional neural network from dlib. These embeddings are stored in a PostgreSQL database using SQLAlchemy ORM. Each user entry includes an ID, name, vector array (ARRAY(Float)), and a base64-encoded facial image.

As shown in Table 2, backend models based on FaceNet (*Facenet512* and *Facenet*) reproduce the claimed LFW accuracy most closely, whereas lightweight architectures such as *OpenFace*, *DeepFace*, and *DeepID* significantly lag under the same evaluation protocol.

A qualitative pros/cons comparison of common face-embedding models is provided in Table 3.

**Figure 1:**
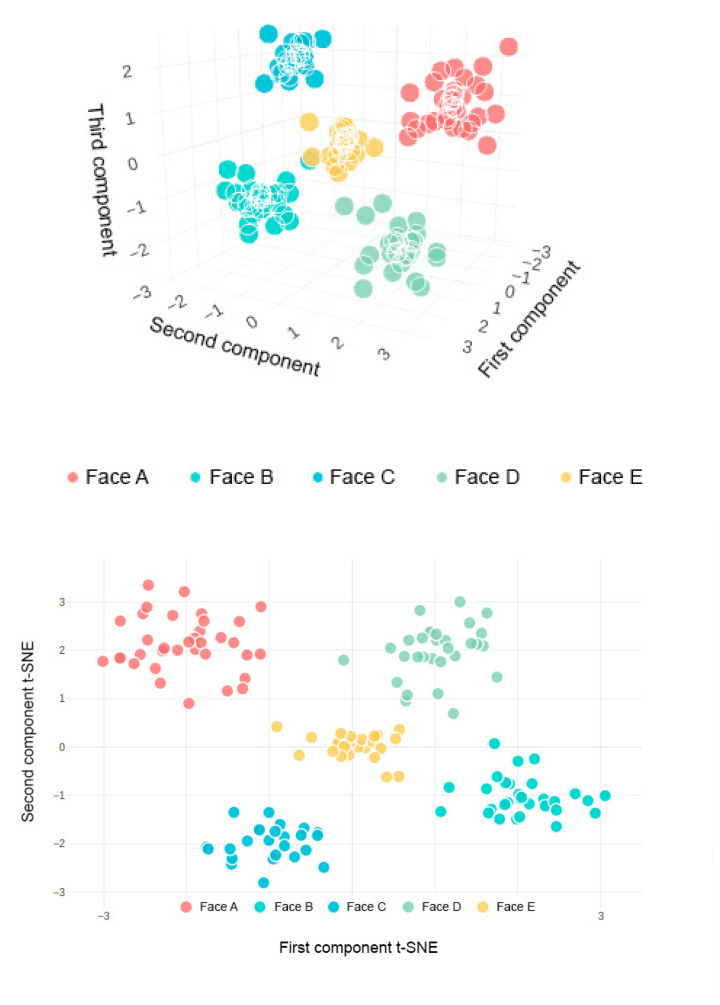
t-SNE *visualization of facial embedding space:* (**top**)—3D map; (**bottom**)—2D projection. Compact, well-separated clusters indicate good class separability and low intra-class variability.

To further illustrate how these quantitative differences translate into the structure of the learned feature space, Figure 1 visualizes the 128-dimensional embeddings using t-SNE: classes formed by high-performing models produce compact, well-separated clusters.

### 3.7. API and Admin Interface

To integrate the recognition module with external services, a RESTful API was developed using the FastAPI framework. The API provides endpoints for user registration, authentication, template deletion, and service-level queries. Following REST principles and using JSON for data exchange, the system ensures compatibility with both lightweight IoT devices and full-scale web applications.

For administrative management, a web-based panel built on SQLAdmin was implemented, providing access to the PostgreSQL database via a browser. The  interface supports standard CRUD operations, log inspection, and system monitoring. Both components (the API and the admin panel) are containerized with Docker, which simplifies deployment, scaling, and portability across server environments and embedded platforms.

### 3.8. Palm Vein Recognition System

The palm vein recognition module is based on near-infrared (NIR) imaging. For experiments, the publicly available Contactless Knuckle, Palm Print and Vein Dataset was used, collected under 850 nm illumination. The dataset contains images of 98 individuals, with 40 samples per subject (20 per hand), for a total of 3920 images. The dataset was divided into training and testing subsets in an 80/20 ratio with subject-wise separation, ensuring that images from the same individual did not appear in both subsets. All images have a resolution of 640 × 480 and are stored in .bmp format.

**Figure 2:**
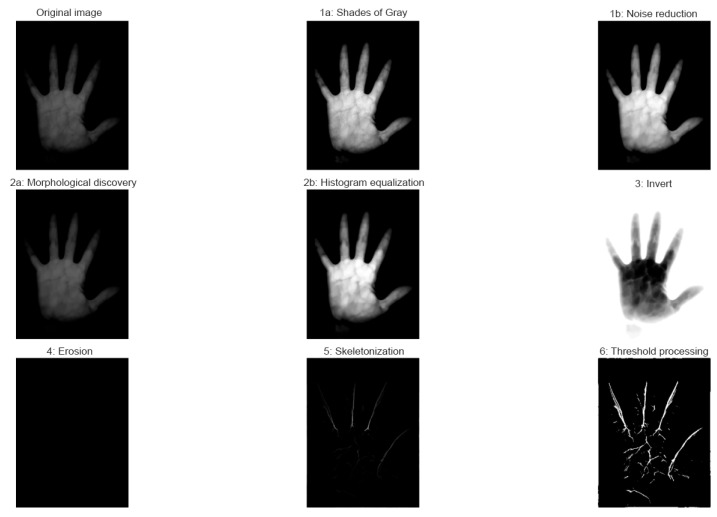
Preprocessing pipeline applied to palm vein region of interest (ROI).

The region of interest (ROI) (Figure 2) of the palm was extracted using the MediaPipe framework, which detected palm landmarks and cropped the corresponding area.

The extracted ROI underwent several preprocessing steps. The first step was noise reduction, where Gaussian blurring or median filtering was applied to remove random pixel fluctuations in NIR images. Noise suppression enhanced the visibility of vein contours and improved the effectiveness of subsequent morphological operations.

The second step involved morphological transformations, including dilation, erosion, opening, and closing. These operations stabilized the geometric structure of the image, merged fragmented vein segments into continuous shapes, and reduced residual noise.

The third stage was binarization and thresholding. At this step, the ROI image was converted into a binary representation: pixels with intensity above a defined threshold were considered foreground, while those below the threshold were identified as part of the venous structure. This enhanced selectivity and prepared the image for further analysis.

The final stage was skeletonization, which reduced the vein thickness to single-pixel-wide lines. Skeletonization produced a geometric map of the vascular structure, serving as the basis for feature vectorization. Additionally, it reduced the information volume of the image, lowering computational costs for the recognition model.

The dataset was divided into training and testing subsets in an 80/20 ratio with subject-wise separation (ensuring that the same individual did not appear in both subsets). As a result, 3136 images were used for training and 784 for testing.

## 4. Results

### 4.1. Face Recognition Performance

The proposed system employed the face_recognition library to extract 128-dimensional face embeddings using a pretrained ResNet-based model. Embedding comparisons were performed using Euclidean distance.

To evaluate the discriminative ability of the extracted features, dimensionality reduction with t-SNE was applied. The 2D projection (Figure 1) revealed well-separated clusters corresponding to different individuals, confirming the reliability of the embedding space for classification.

### 4.2. Stereo-Based Anti-Spoofing

Face liveness detection methods leveraging stereo vision have been validated in recent studies through disparity map analysis and texture-based approaches [21,22].

**Figure 3:**
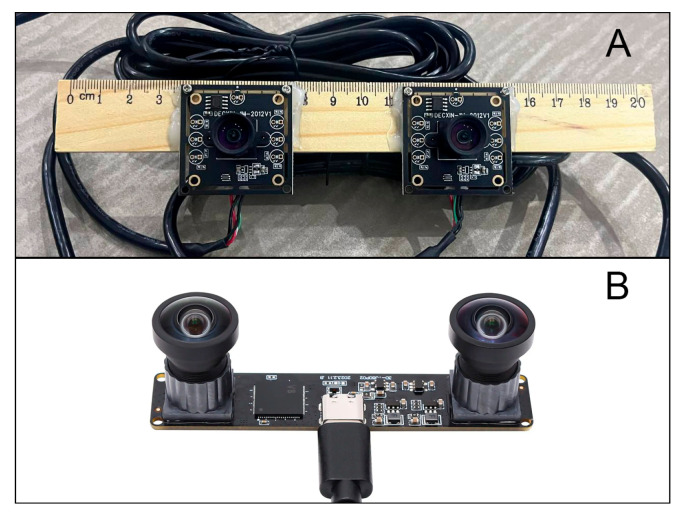
Stereo setup with (**A**) two USB cameras and (**B**) a 7 cm baseline for disparity estimation.

**Figure 4:**
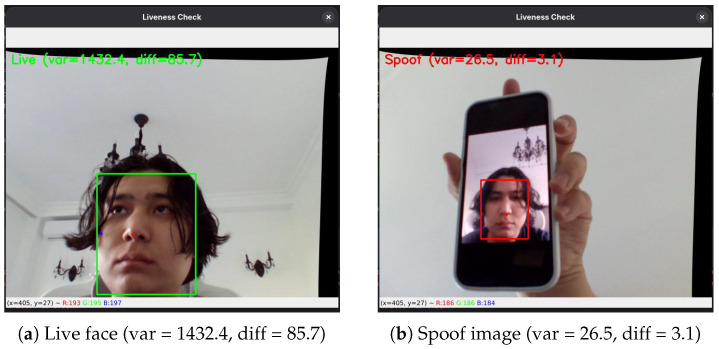
Stereo-based liveness detection results. (**a**) Live face with high disparity variance and nose-cheek depth difference. (**b**) Spoof image printed photo.

A stereo setup consisting of two USB cameras with a 7 cm baseline was configured to estimate depth via disparity map construction (Figure 3). The variance for live faces averaged 1432.4, whereas spoofing attempts (e.g., printed photos or screens) exhibited a variance of only 26.5. The mean pixel difference between the nose and cheek regions was 85 px for genuine faces and 3.1 px for spoofed ones (Figure 4).  

**Figure 5:**
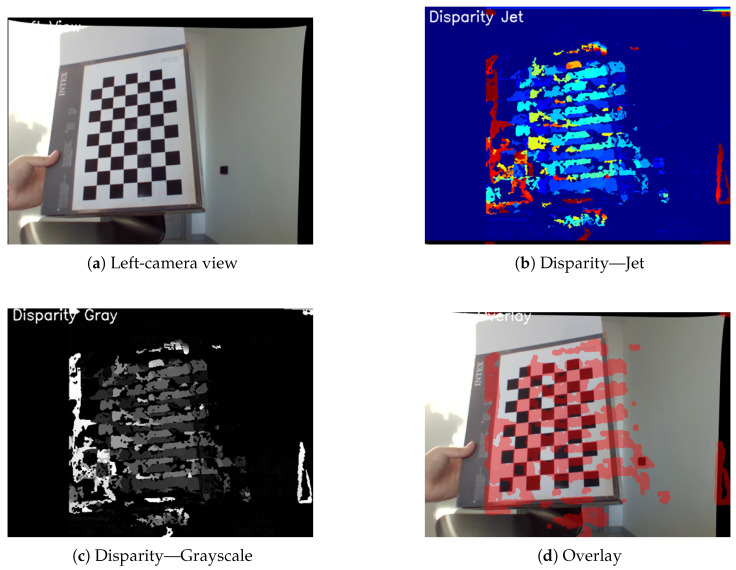
Left-camera view (**a**) and three visualizations of disparity maps: (**b**) Jet colormap, (**c**) grayscale, and (**d**) overlay.

Figure 5a shows the left-camera view of the calibration target. Disparity maps were visualized in three formats: jet colormap (Figure 5b), grayscale (Figure 5c), and overlay (Figure 5d), enhancing depth analysis and spoof detection interpretability. Integrating stereo-based liveness detection significantly improves system security against spoofing attacks. Beyond higher detection accuracy, depth representations provide an intuitive diagnostic tool, enabling engineers to quickly calibrate the camera baseline and block-matching parameters on-site. Such visual feedback shortens deployment time and helps maintain stable performance under system relocation or changing illumination conditions.

### 4.3. Palm Vein Classification Performance

The palm vein recognition system utilized preprocessed near-infrared (NIR) images at a wavelength of 850 nm. A total of 3920 images were collected from 98 subjects and split into 80% for training and 20% for testing. The region of interest (ROI) was automatically extracted using MediaPipe, while subsequent preprocessing stages included noise reduction, morphological operations, binarization, and skeletonization.

**Figure 6:**
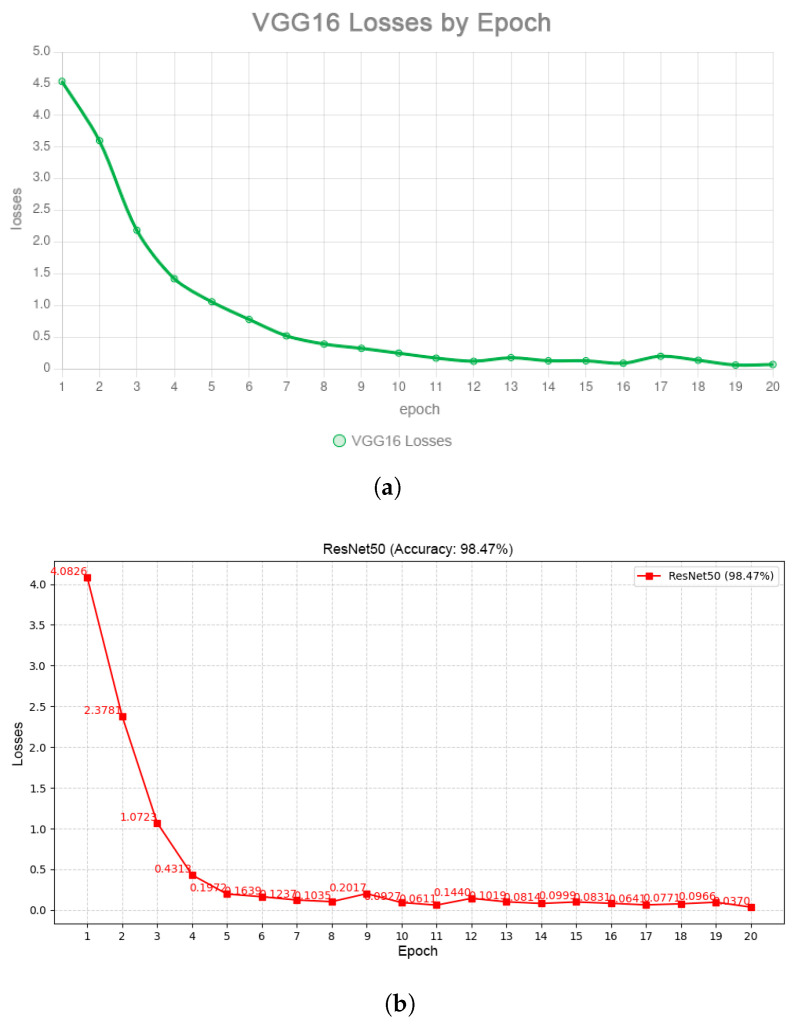
Training loss per epoch. (**a**) ResNet-50 loss dynamics. (**b**) VGG-16 loss dynamics.

Two convolutional neural network (CNN) architectures were evaluated: ResNet50 and VGG16. The ResNet50 model achieved a classification accuracy of 98.34% after 20 training epochs, outperforming VGG16, which reached 89.74% over the same number of epochs. The superiority of ResNet50 is attributed to its deeper architecture and residual learning capabilities (Figure 6a,b). Furthermore, a stratified cross-validation experiment confirmed the robustness of the obtained results. Across five folds, ResNet50 maintained an average accuracy of 97.9% (±0.6), whereas VGG16 exhibited substantially higher variability (88.7 ± 1.8%). In addition to overall accuracy, precision, recall, and F1-score were tracked for each class. The metrics for ResNet50 consistently exceeded 0.97, indicating that the  model did not rely on class imbalance but reliably captured discriminative vein patterns across all subjects. These findings demonstrate that deeper residual connections not only accelerate convergence but also provide a more stable vector representation that generalizes effectively to previously unseen data. This property is crucial for real-world scenarios such as access control turnstiles or e-KYC platforms.

### 4.4. Training Models with Triplet Loss on ResNet50

**Figure 7:**
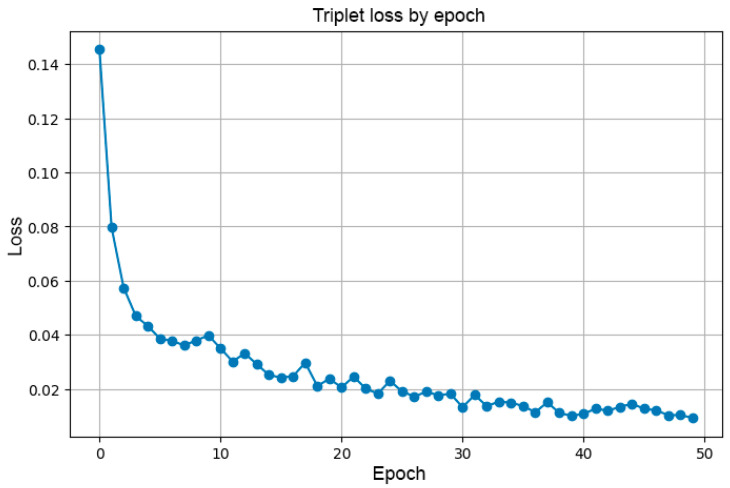
Triplet-loss convergence during embedding training.

The ResNet-50 backbone was fine-tuned for 50 epochs using the Triplet Loss objective with a batch-all approach and online hard-negative mining. The training setup employed a learning rate of $1\times 10^{-4}$ (decreased every 10 epochs), a batch size of 32, and a margin parameter of 0.2. Under these conditions, each palm vein ROI (Figure 2) was mapped into a 128-dimensional embedding space, where intra-class distances were minimized and inter-class distances were maximized. Figure 7 illustrates the smooth convergence of the loss function, while Table 3 summarizes the statistical properties of the embedding space.

As shown in Table 4, the embedding space *evolves consistently* and in the desired direction during 50 epochs of fine-tuning. The intra-class distance decreases by more than 56% (from 0.92 to 0.40), indicating that samples of the same subject are brought significantly closer together. Conversely, the inter-class distance increases by approximately 45% (from 1.11 to 1.60), demonstrating that embeddings of different subjects are pushed further apart. These changes result in a steady reduction of the Triplet Loss value—from 0.14 at epoch 0 to 0.01 at epoch 50—confirming that the network progressively learns a highly discriminative feature space.

In practice, *such separation enables more reliable identification from a single sample*: new users can be enrolled with only a few reference images while maintaining a low false-match rate, since their embeddings remain outside the compact clusters of existing identities. Moreover, the results suggest that employing more aggressive hard-negative mining strategies or moderate data augmentation (e.g., photometric noise or affine transformations) could further increase the inter-class margin without substantially prolonging training time.

Overall, the findings demonstrate that ResNet-50 trained with Triplet Loss produces a robust and well-structured embedding space, making it particularly suitable for biometric applications such as palm vein recognition.

However, to achieve even higher performance and improve the generalization ability of the model, it would be advisable to train on a larger and more diverse dataset. This would enable the system to better adapt to variations and conditions, which is critical for real-world deployment scenarios.

### 4.5. System Integration and Deployment

To make the system structure explicit, we summarize all modules and their connections. The solution consists of four layers:

- Face recognition module: embedding generation with a 128D ResNet-based network (with normalization) and stereo-based passive liveness detection.
- Palm-vein module: palm ROI extraction (segmentation/enhancement) followed by CNN-based classification of 850 nm NIR images.
- Backend: FastAPI REST endpoints with JWT authentication; PostgreSQL storage; admin interface via SQLAdmin.
- Deployment layer: Dockerized services on an AWS instance for portable, scalable rollout.

**Figure 8:**
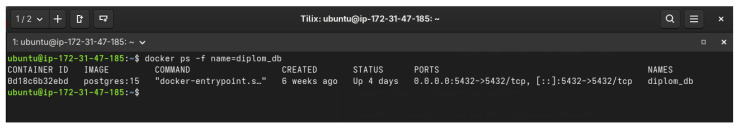
Launching a PostgreSQL container on AWS.

**Figure 9:**
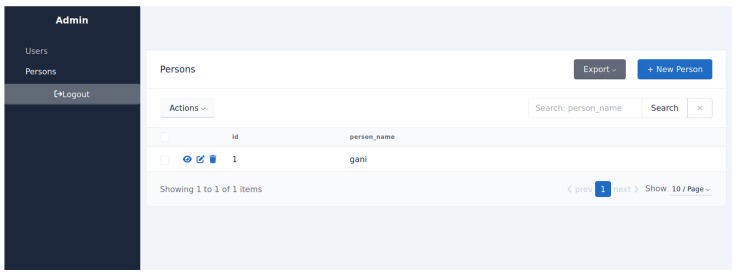
SQLAdmin management panel with registered users.

The system was deployed within a Docker container, while a PostgreSQL database was hosted on an AWS server (Figure 8). This configuration ensured both portability of the recognition service and reliability of data storage. The FastAPI framework handled API requests for embedding extraction, user registration, and verification, with REST endpoints secured by JWT-based authentication. A web interface built on SQLAdmin provided convenient access to the PostgreSQL database for managing biometric records and inspecting logs (Figure 9). Such an architecture simplified system administration and facilitated deployment both on server hardware and embedded platforms.

**Figure 10:**
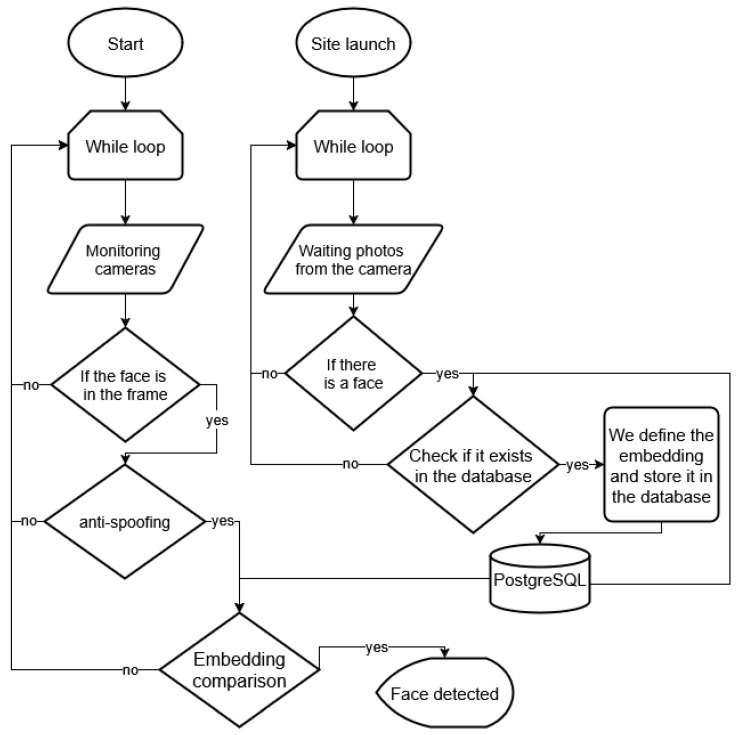
Real-time processing pipeline of the proposed biometric system: the service continuously captures camera frames, applies anti-spoofing, derives embeddings, and verifies them against the PostgreSQL database.

**Figure 11:**
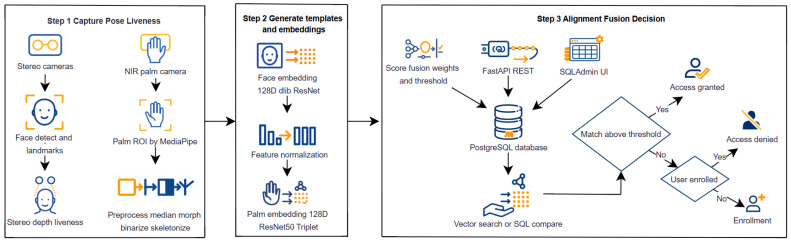
Architecture of the proposed biometric system.

Figure 10 illustrates the real-time processing pipeline, and Figure 11 shows the overall system architecture and inter-module data flow.

The overall architecture demonstrated real-time processing capabilities, high classification accuracy, and strong resistance to spoofing attacks, making it suitable for deployment in *eKYC*, access control, and smart security systems.

Figure 11 illustrates the overall architecture of the proposed biometric system, which integrates three main stages: (1) capture and liveness detection using stereo and NIR cameras for face and palm imaging; (2) embedding generation through deep neural networks (128D ResNet-based models with normalization); and (3) fusion-based decision using score alignment, FastAPI-PostgreSQL backend, and SQLAdmin interface for enrollment and access control. This modular design ensures real-time operation, spoof resistance, and scalability on edge IoT devices.

## 5. Discussion

Recent reviews and comparative studies highlight the strengths and weaknesses of various biometric modalities, highlighting the benefits of multimodal and subdermal biometrics in real-world conditions [23,24]. Although palm vein recognition is often considered a secure biometric modality, contemporary research has shown that it is not entirely resistant to presentation attacks. In particular, Tome and Marcel [5] showed that spoofing palm vein systems is feasible using printed or synthetic artifacts under certain conditions.

From an optimization perspective, we explicitly balance accuracy, convergence speed, and model complexity. Compared to VGG16, the residual backbone converges faster and generalizes more stably (Section 4.3), while Triplet Loss fine-tuning yields a more discriminative embedding space (Section 4.4). On the deployment side, a lightweight, Dockerized FastAPI service with JWT minimizes overhead and simplifies scaling to edge targets (Section 4.5), aligning the pipeline with constrained-device requirements.

This study presents a comprehensive approach to biometric identification based on palm vein recognition using infrared imaging and convolutional neural networks. The proposed system addresses several key limitations of traditional biometric methods, particularly those relying on facial or fingerprint recognition, by shifting the focus to subsurface anatomical features that are more secure and harder to replicate.

The obtained results clearly demonstrate the effectiveness of combining infrared palm vein imaging, ROI detection with MediaPipe, and image enhancement techniques. The preprocessing pipeline, which included noise suppression, morphological operations, binarization, and skeletonization, significantly improved the visibility of venous structures. This directly influenced model classification quality, as clearer anatomical features enabled convolutional layers to extract more informative spatial patterns.

**Figure 12:**
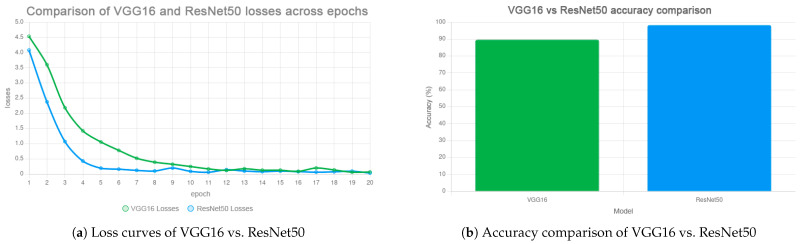
Performance comparison. (**a**) Loss curves of VGG16 vs. ResNet50. (**b**) Accuracy comparison over epochs.

When comparing classification performance, the ResNet50 architecture outperformed VGG16 both in terms of accuracy and convergence speed. Specifically, ResNet50 achieved an accuracy of 98.34% after 20 training epochs, whereas VGG16 stabilized at 89.74% after the same number of epochs. This result underscores the advantage of deeper residual networks in capturing complex biometric patterns, which is particularly important for analyzing subsurface venous structures with subtle yet distinctive characteristics (see Figure 12).

The system achieves 20–25 fps inference on edge-class GPU, demonstrating its suitability for real-time applications with constrained resources. This performance ensures the feasibility of deploying the system on edge devices, providing low-latency and efficient processing, which is critical for real-time biometric applications.

### Computational Complexity

The time complexity of the training process is O(n·m·k), where:

- *n* is the number of samples;
- *m* is the number of features;
- *k* is the number of parameters in the model.

Furthermore, the use of the Contactless Knuckle, Palm Print and Vein Dataset provided a reliable and diversified foundation for training. Despite certain limitations, such as a relatively small sample size, uniform lighting conditions, and limited demographic diversity, this dataset enabled meaningful prototyping and validation. Importantly, the separation of training and test sets without subject overlap ensured a fair assessment of the model’s generalization capability—an essential requirement for biometric systems deployed in real world scenarios.

In the context of prior research, the obtained results are consistent with the literature, which emphasizes the superiority of palm vein biometrics in terms of resistance to attacks, robustness under varying conditions, and long-term reliability. However, this work also highlights the need to expand datasets with greater demographic and environmental diversity, as well as to integrate multimodal approaches (e.g., combining palm vein and facial embeddings) to further enhance accuracy.

Several industry solutions have already demonstrated the feasibility of integrating biometric authentication with edge computing technologies and IoT platforms [25,26]. Palm vein biometrics, in particular, is being explored for deployment in medical systems and access control applications [25].

Finally, the architecture based on FastAPI, Docker, SQLAlchemy, and SQLAdmin provides a modular and scalable backend infrastructure suitable for deployment in secure environments such as universities, government institutions, and IoT-based access control systems.

## 6. Conclusions

The growing demand for secure and automated identification systems underscores the importance of integrating biometric technologies with advanced solutions in artificial intelligence (AI) and the Internet of Things (IoT). This work presented the development of an intelligent biometric recognition platform that combines facial and palm vein modalities to provide robust multi-level authentication.

A comprehensive review of relevant technologies in AI, IoT, and biometrics was conducted, with particular emphasis on the applicability of different physiological traits, such as facial features and subcutaneous venous structures. The proposed system leverages AI-based embeddings and IoT infrastructure to enable real-time operation, high accuracy, and resilience against presentation attacks.

Face recognition was implemented using a classifier-free approach based on the face_recognition library, augmented with stereo vision for liveness detection. The back-end was built with FastAPI and PostgreSQL, containerized with Docker, and deployed in a cloud infrastructure. The system achieved a recognition accuracy of 98.7%, with a false acceptance rate (FAR) of 1.2% and a false rejection rate (FRR) of 2.6%. The quality and distribution of embeddings were validated through t-SNE visualization. The implementation was carried out in Python 3.13 using OpenCV 4.10.0.84 for image processing, face_recognition 1.3.0 for facial feature extraction, and PyTorch 2.7.X for training deep learning models, ensuring reproducibility, compatibility, and high performance of the developed algorithms.

For palm vein recognition, near-infrared (NIR) imaging was employed. The images underwent preprocessing steps including automatic ROI selection, filtering, morphological operations, thresholding, and skeletonization. At this stage, two deep learning models— VGG16 and ResNet50—were trained for the classification task, achieving accuracies of 89.74% and 98.34%, respectively. Additionally, an experiment was conducted by fine-tuning ResNet50 with the Triplet Loss function. This enabled the formation of more reliable and well-structured features, as confirmed by the embedding space visualization. Such an approach makes the model particularly suitable for biometric applications. However, it should be noted that its full potential can only be realized when trained on a larger and more diverse dataset, which remains an important direction for future research.

The proposed system demonstrates strong potential for deployment in real-world scenarios such as campus access control, identity verification, and biometric authentication on edge devices. Its architecture supports modularity and scalability, enabling seamless integration into both cloud-based and edge environments. Recent studies further confirm the trend toward edge-deployed AI-driven biometric systems with built-in liveness detection and privacy-preserving mechanisms [20,27].

Overall, the proposed approach provides several key advantages: the integration of surface-level and subcutaneous biometric features for enhanced security, a flexible infrastructure that supports autonomous operation, and the use of embedding-based representations that eliminate the need for traditional classifiers. At the same time, limitations related to insufficient dataset diversity, controlled laboratory conditions, and the absence of comprehensive data protection mechanisms must be addressed in future work.

## Figures and Tables

**Table 1:** Classification of stereo calibration quality based on RMS reprojection error.

RMS Error, px	Quality Assessment	Typical Application
<0.3	High accuracy	Laboratory metrology
0.3–0.7	Very good	Robotics, AR
0.7–1.2	Acceptable	Stereo measurements, SLAM
>1.5	Poor	Raw 3D modeling; requires refinement

**Table 2:** Comparison of reported and reproduced model accuracy on the LFW benchmark dataset.

Model	Measured Accuracy, %	Reported Accuracy, %
Facenet512	98.4	99.6
Human-beings ^1^	97.5	97.5
Facenet	97.4	99.2
Dlib	96.8	99.3
VGG-Face	96.7	98.9
ArcFace	96.7	99.5
GhostFaceNet	93.3	99.7
SFace	93.0	99.5
OpenFace	78.7	92.9
DeepFace	69.0	97.3
DeepID	66.5	97.4

^1^ “Human-beings” denotes the human baseline performance reported in the LFW benchmark.

**Table 3:** Pros/cons of common face-embedding models in the context of edge-oriented deployments.

Aspect	FaceNet	ArcFace	VGG-Face	MobileFaceNets
Objective/loss	Metric learning with *triplet loss*; typically 128-D embeddings	Additive angular-margin softmax; typically 512-D embeddings	Softmax classification (VGG-16 backbone)	Mobile-optimized backbones; classification/metric variants (128–256-D)
Accuracy (typical)	Strong baseline; sensitive to mining quality	State-of-the-art on many benchmarks	Lower than modern residual models	Lower than heavy models; competitive per FLOP
Model size/ compute	Moderate (depends on backbone)	Higher with large backbones (e.g., ResNet-100)	Heavy (VGG-16; large memory footprint)	Very small; depthwise separable convolutions
Convergence/ training	Requires careful triplet mining; can converge slower	Fast, stable convergence	Stable but prone to overfitting on limited data	Fast; designed for constrained training/inference
Edge suitability	Good trade-off with medium backbones	High accuracy; edge-suitable after pruning/quantization	Less suitable due to size/memory	Highly suitable for mobile/edge devices
Availability	Widely available pretrained implementations	Widely available pretrained weights	Pretrained (older) models readily available	Pretrained mobile variants available

**Table 4:** Embedding space statistics across training epochs.

Metric	Epoch 0	Epoch 25	Epoch 50
Intra-class distance (μ±σ)	0.92 ± 0.11	0.46 ± 0.07	0.40 ± 0.05
Inter-class distance (μ±σ)	1.11 ± 0.13	1.55 ± 0.12	1.60 ± 0.10
Triplet Loss	0.14	0.02	0.01

## Data Availability

The source code and trained model of the proposed biometric identification system are openly available in the GitHub repository: GitHub, https://github.com/gans-1337/palm-src (accessed on 26 October 2025).
